# Strain-induced growth instability and nanoscale surface patterning in perovskite thin films

**DOI:** 10.1038/srep26075

**Published:** 2016-05-19

**Authors:** Shishir Pandya, Anoop R. Damodaran, Ruijuan Xu, Shang-Lin Hsu, Joshua C. Agar, Lane W. Martin

**Affiliations:** 1 Department of Materials Science and Engineering, University of California, Berkeley, Berkeley, CA 94720, USA; 2 Materials Science Division, Lawrence Berkeley National Laboratory, Berkeley, CA 94720, USA.

## Abstract

Despite extensive studies on the effects of epitaxial strain on the evolution of the lattice and properties of materials, considerably less work has explored the impact of strain on growth dynamics. In this work, we demonstrate a growth-mode transition from 2D-step flow to self-organized, nanoscale 3D-island formation in PbZr_0.2_Ti_0.8_O_3_/SrRuO_3_/SrTiO_3_ (001) heterostructures as the kinetics of the growth process respond to the evolution of strain. With increasing heterostructure thickness and misfit dislocation formation at the buried interface, a periodic, modulated strain field is generated that alters the adatom binding energy and, in turn, leads to a kinetic instability that drives a transition from 2D growth to ordered, 3D-island formation. The results suggest that the periodically varying binding energy can lead to inhomogeneous adsorption kinetics causing preferential growth at certain sites. This, in conjunction with the presence of an Ehrlich-Schwoebel barrier, gives rise to long-range, periodically-ordered arrays of so-called “wedding cake” 3D nanostructures which self-assemble along the [100] and [010].

Next-generation thin-film devices will require the production of ultrathin films and superlattices with atomically-sharp interfaces and, potentially, self-patterned 3D nanostructures to enable a range of applications^1^,^2^,^3^,^4^. In turn, the ability to control and manipulate the nature of growth processes in epitaxial films, for instance between 2D-growth modes for planar structures to self-organized 3D nanostructures, is critical. In order to achieve such control, an understanding of the physics of the complex atomistic processes that occur at the growth-front is essential. Such control can be obtained by understanding the kinetics of growth instabilities in non-equilibrium growth processes. For example, in the growth of Pt/Pt (111)^5^ and Ag/Ag (111)^6^, multi-terrace islands can arise as a consequence of limited interlayer mass transport kinetics due to an Ehrlich-Schwoebel (ES) energy barrier which restricts the diffusion of adatoms down a step edge and biases growth towards multi-terrace islands^7^,^8^. Quantitative estimates of this barrier have been extracted from scanning tunneling microscopy (STM) studies^5^,^9^ and kinetic Monte-Carlo^10^ and phase-field simulations^11^. The ES barrier can also cause a morphological Bales-Zangwill instability^12^, which is diffusional in origin and promotes waviness of the vicinal terrace edge^13^ and can destabilize step-flow growth producing step meandering and multi-terrace island formation^14^.

At the same time, research has suggested that the growth kinetics of heteroepitaxial thin-film systems can be strongly dependent on epitaxial strain. In both metal^15^,^16^ and semiconductor^17^ systems, the diffusion of adatoms can be strongly influenced by the surface strain. For instance, the activation energies for Ag self-diffusion can vary greatly depending on the strain state with considerably lower values being observed for diffusion on a strained, pseudomorphic Ag monolayer on Pt (111) (60 meV) as compared to unstrained Ag (111) surfaces (97 meV)^6^. Additionally, relaxation of epitaxial strain via misfit dislocation formation can also affect surface diffusion and nucleation. Again, in Ag/Pt (111) heterostructures, after two monolayers of Ag are deposited, the large lattice mismatch (4.3%) is accommodated by the formation of stress relief patterns where the misfit dislocations on the surface repel the adsorbed adatoms and partition them into 3D nanostructures^18^. In Si_1−x_Ge_x_ films on Si (001), strain relief manifests in a series of morphological changes. At submonolayer coverages surface reconstruction begins to form^19^,^20^. Later, 3D islands with well-defined facets that are coherent with the substrate lattice begin to form. The driving force for such facet formation is the effective strain relief along the crystallographic direction that compensates the increase in surface energy^21^. Strain relief can also manifest by cross-hatching of the surface or formation of undulations or “ripples.” Such structures have been explained by equilibrium calculations as the result of balancing the strain energy from the buried misfit dislocations and the surface energy^22^,^23^ and by incorporating strain relaxation due to dislocation glide in the film interior and lateral surface transport that eliminates surface steps^24^,^25^.

On the other hand, the evolution of surface morphology in the growth of complex-oxide thin films has been studied far less. The limited work includes experimental study of a growth mode transition from layer-by-layer to step-flow growth in SrRuO_3_/SrTiO_3_ (001) heterostructures^26^ and theoretical study of the evolution of growth mode under competing elastic and kinetic forces^27^. Recently, researchers have reported evidence of surface cross-hatch morphologies in SrTiO_3_/NdGaO_3_ (110) heterostructures^28^ which were attributed to anisotropic strain relaxation with 60° misfit dislocation formation that caused lateral surface step flow. On the other hand, in La_0.5_Ca_0.5_MnO_3_/SrTiO_3_ (001) heterostructures^29^, it was suggested that the interaction of the depositing adatoms with the dislocation stress field was responsible for the cross-hatching.

Building off of this work in metals, semiconductor, and oxide thin films, the current work demonstrates that the presence of misfit dislocations at a buried interface produce a periodic, modulated strain field that alters the adatom binding energy landscape and leads to a kinetic instability that drives a transition from 2D- to ordered, 3D-island growth in PbZr_0.2_Ti_0.8_O_3_/SrRuO_3_/SrTiO_3_ (001). The result is long-range, periodic, ordered arrays of “wedding cake” 3D nanostructures along the in-plane [100] and [010]. The spatially inhomogeneous surface strain state drives deterministic changes in the surface morphology and potentially provides a useful experimental pathway to understand and control the dynamics of epitaxial thin-film growth processes.

## Results

**Figure 1:**
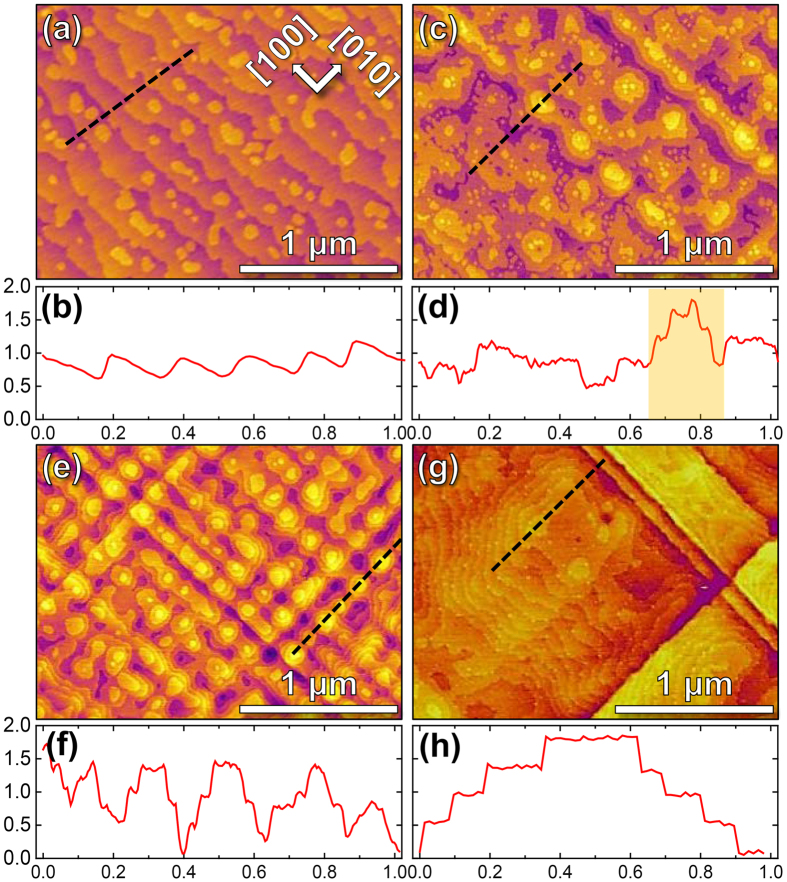
Atomic force microscopy (AFM) images and extracted line profiles (black dashed line in AFM image) for heterostructures with PbZr_0.2_Ti_0.8_O_3_ thickness of (**a,b**) 25 nm, (**c,d**) 40 nm, (**e,f**) 55 nm, and (**g,h**) 150 nm, respectively. For (**b,d,f,h**), the vertical scale is in units of nanometers and the horizontal scale in units of microns.

Details of the film growth process are provided in the Methods. Atomic force microscopy studies of surface topography reveal that 25 nm thick heterostructures (Fig. 1a) exhibit atomically-smooth, terraced surfaces indicative of growth in a predominantly step-flow growth mode with some formation of 2D islands on the flat terraces which eventually coalesce with the vicinal step edges. A line-trace across this surface confirms that the individual steps are a single unit cell in height (Fig. 1b). As the thickness of the film increases to 40 nm (Fig. 1c), a change in the surface structure occurs whereby localized multi-terrace islands, which are ordered along [100] and [010], are observed to form (the multi-layer island formations can be seen in the line profile, Fig. 1d). By a film thickness of 55 nm (Fig. 1e), a highly-ordered array of multi-terrace islands is observed (confirmed by the line trace, Fig. 1f). The ordered nature of the multi-terrace islands gives the appearance of a cross-hatched or ripple-like pattern that runs along the [100] and [010]. We have conducted detailed analysis of the linear density of these features by measuring several areas of the same size over several areas on several different heterostructures of each thickness. The linear density of the cross-hatched or ripple-like features increases from an average of 0.78 μm^−1^ for 40 nm thick films to an average of 4.53 μm^−1^ for 55 nm thick films. When the heterostructure thickness reaches 150 nm (Fig. 1g), the multi-terrace islands are reminiscent of so-called “wedding-cake” structures (due to their uncanny resemblance)^30^ with a large number of terraces (Fig. 1h). The tilted structure of ferroelastic domains in the PbZr_0.2_Ti_0.8_O_3_^31^ can also be seen superimposed on the multi-terrace island structure (Fig. 1g).

These multi-terrace islands in the PbZr_0.2_Ti_0.8_O_3_ heterostructures bear a striking resemblance to the features observed in homoepitaxial metal films^5^,^6^. Again, in metal films, such features were explained solely by the presence of an ES barrier which limits interlayer mobility. Despite some similarities, there are a few key differences between the observations in the PbZr_0.2_Ti_0.8_O_3_ heterostructures as compared to that in the metal films. First, we note that multi-terrace island formation does not occur in the PbZr_0.2_Ti_0.8_O_3_ heterostructures until a film thickness of 30–40 nm as opposed to the onset in sub-monolayer deposition for metal films^5^. Second, in contrast to metal systems where there is no long-range order to the island formation, the multi-terrace islands in PbZr_0.2_Ti_0.8_O_3_ are found to be aligned along the [100] and [010] and to occur in bands with an average periodicity that scales inversely with the film thickness.

**Figure 2:**
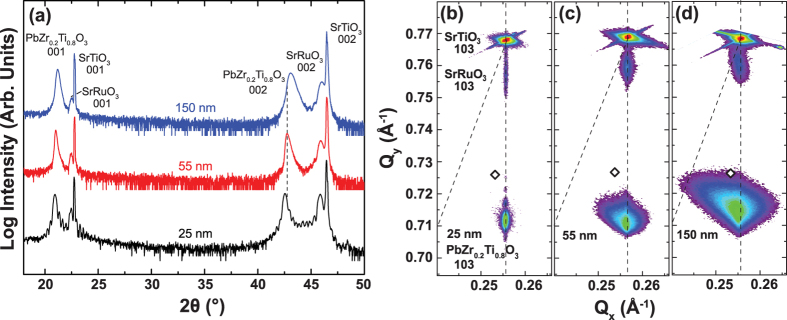
(**a**) θ–2θ X-ray diffraction patterns for various thin film heterostructures reveal single-phase and epitaxial films. Asymmetric reciprocal space mapping studies about the 103-diffraction conditions of heterostructures with PbZr_0.2_Ti_0.8_O_3_ thicknesses of (**b**) 25 nm, (**c**) 55 nm, and (**d**) 150 nm. Diamond symbols (black, open) represent the bulk, fully-relaxed film peak position.

To understand the mechanism underlying both the delayed onset and ordering of these multi-terraced island features, we probed the structure of the films using X-ray diffraction. θ–2θ diffraction patterns reveal that all films are single-phase and epitaxial (Fig. 2a). As the film thickness increases from 25 nm to 150 nm, the 002-diffraction peak for the PbZr_0.2_Ti_0.8_O_3_ shifts to higher Bragg angles indicating a decrease in the out-of-plane lattice parameter possibly due to strain relaxation. Further insight is gained from reciprocal space maps (RSM) about the 002- and 103-diffraction conditions. Symmetric RSMs about the 002-diffraction condition confirm that the morphological features observed herein are not the result of ferroelastic domain formation (data not shown for brevity). Asymmetric RSMs about the 103-diffraction condition reveal that the strain in the films steadily relaxes with increasing film thickness (Fig. 2b–d). It can be seen in the thinnest PbZr_0.2_Ti_0.8_O_3_ films (25 nm, Fig. 2b) that the PbZr_0.2_Ti_0.8_O_3_, the SrRuO_3_, and the SrTiO_3_ substrate are all found to possess the same Q_x_ values for their diffraction peaks which is indicative of coherently strained films with in-plane lattice parameters that match that of the substrate. As the film thickness is increased (*i.e.*, 55 nm and 150 nm, Fig. 2c,d, respectively), however, the diffraction peak for the PbZr_0.2_Ti_0.8_O_3_ is found to exhibit intensity that is smeared to smaller Q_x_ values revealing that the in-plane lattice parameter of the film is larger than that of the substrate and the bottom electrode and that the film has partially relaxed towards its bulk in-plane lattice constant. It should also be noted that while SrRuO_3_ bottom electrodes of identical thickness were grown under identical growth conditions, there is smearing of the Q_x_ values of SrRuO_3_ with increasing thickness of PbZr_0.2_Ti_0.8_O_3_ film indicating lattice distortion in the SrRuO_3_ layer possibly as a result of strain relaxation of the PbZr_0.2_Ti_0.8_O_3_ film.

To quantify the magnitude of strain relaxation, we calculated an average diffraction peak position (θ_avg_, ω_avg_) weighted by the intensity. The effective in-plane lattice parameter *a*_||_ and in-plane strain relaxation can thus be calculated as


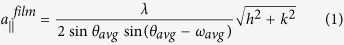



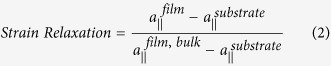


**Figure 3:**
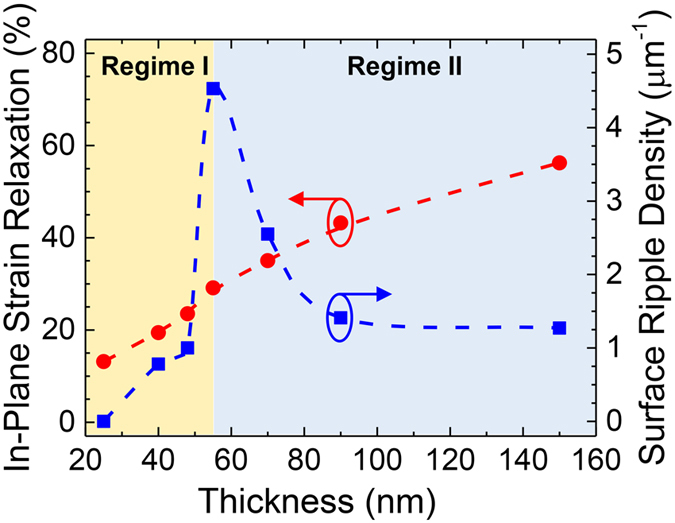
In-plane strain relaxation (%, red circles) and surface ripple density (μm^−1^, blue squares) as a function of film thickness.

In turn, it can be seen that the PbZr_0.2_Ti_0.8_O_3_ film monotonically relaxes with increasing film thickness (red data, Fig. 3). The surface ripple density (blue data, Fig. 3), however, first increases trending similarly to the strain relaxation in Regime I-before abruptly peaking and then rapidly decreases with a subsequent increase in the film thickness (thus trending counter to the strain relaxation in Regime II).

**Figure 4:**
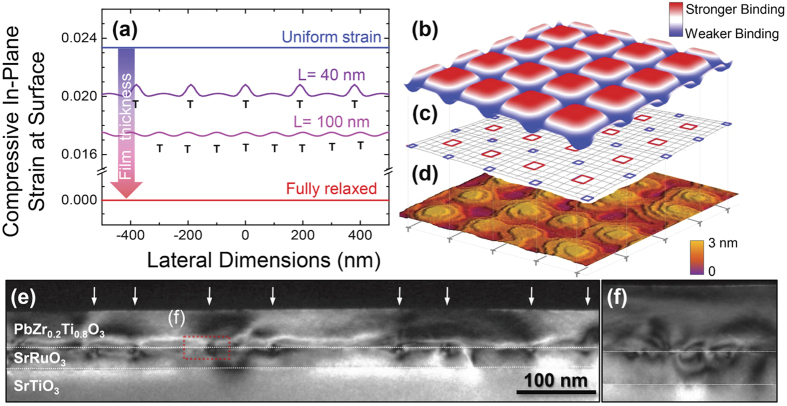
(**a**) Calculated variation of compressive in-plane strain at the film surface as a function of film thickness. The data, from top to bottom, represent the case for pseudomorphically-strained (blue), 40 nm thick (purple), 100 nm thick (magenta), and fully-relaxed (red) heterostructures and show periodic strain modulations on the surface of 40 nm and 100 nm thick films due to the buried misfit dislocation array (T-symbol). (**b**) Schematic illustration of the variation in the adatom binding energy on the surface-red and blue areas are regions with stronger and weaker binding energy, respectively. (**c**) Schematic illustration of the in-plane lattice parameters on the surface of a film under a partially relaxed biaxial strain-red and blue areas are under less and more compressive strain, respectively. (**d**) The measured topography of a 55 nm thick PbZr_0.2_Ti_0.8_O_3_ heterostructure showing aligned wedding-cake structures. (**e**) Cross-section, dark-field TEM image of the same heterostructure taken under the g = (200) two beam condition along the [012] zone axis showing the presence of dislocations (white arrows point to dislocations located at the PbZr_0.2_Ti_0.8_O_3_/SrRuO_3_ interface) and corresponding strain fields. (**f**) Zoom-in image of the strain-induced contrast from the dislocation strain fields.

Strain relaxation in lattice mismatched heterostructures is usually accommodated by the formation of misfit dislocations during growth^32^,^33^. We propose that the growth instability and formation of spatially-ordered islands is the result of the stress fields from a 2D array of edge dislocations at a buried interface and the subsequent preferential nucleation of adatoms driven by inhomogeneous surface strains^34^,^35^. Let us consider an array of uniformly-spaced, semi-infinite edge dislocations running parallel to and at a distance *l* from the free surface of a semi-infinite body. For this system, the Burgers vector *b* = *a* <100>^36^,^37^,^38^. The in-plane stress at an arbitrary point can be calculated by summing the contributions from all the dislocations in the array. The normal in-plane stress *σ*_*xx*_ along the free surface is given by^39^


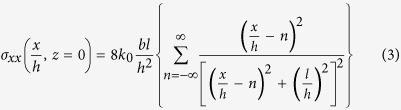


where *z* = 0 implies the stress at the film surface and *x* is an arbitrary point in the planar space, 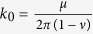 where *μ* and *ν* are the shear modulus and Poisson ratio, respectively, *h* is the dislocation periodicity, *b* is the magnitude of the Burgers vector, and *l* is the distance between the free surface and the dislocation array. The total stress at the film surface is thus calculated by superimposing the compressive stress imposed by the epitaxial strain and that due to the presence of a dislocation array at the interface as calculated above (Eq. 3). The resultant in-plane surface strain can thus be plotted (Fig. 4a).

It can be seen that the pseudomorphically-strained heterostructures exhibit no strain inhomogeneity. As the film thickness increases and the heterostructures begins to relax, however, two effects are observed: 1) the mean compressive strain reduces due to an average strain relaxation and 2) a surface strain modulation due to the periodic strain fields from the buried dislocation network is formed. It can also be seen that in thinner films, the amplitude of strain modulation is larger in comparison to the thicker films. Such modulations in the surface strain state can affect the binding energy of the adatoms. Within linear elastic theory, the binding energy varies linearly with external strain *ε*^*ext*^ and can be written as 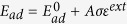, where 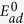 is the binding energy on an unstrained surface, *A* is the surface area, and *σ* is the surface stress tensor induced by the adatom^40^. This difference in the binding energy due to external strain can be understood as the additional elastic energy equal to the work done by the adatom-induced stress against the applied strain. In the present case, relaxation of a film by the formation of buried dislocations at the film-substrate interface causes strain modulation in two orthogonal directions resulting in an inhomogeneous deformation of the surface creating local regions of maximum and minimum biaxial compressive strain (depicted in blue and red respectively, Fig. 4c). This inhomogeneity in the surface strain locally alters the binding energy of the adatoms and creates an inhomogeneous potential energy landscape (Fig. 4b). Quantification of this modulation of the local binding energy is possible, but difficult. For instance, first-principles calculations have been used to examine the strain dependence of adatom binding energies in both homo- and heteroepitaxial growth of single-component metal and semiconductor systems^15^,^40^. Such quantification for the current multi-component system under study in this work, however, has not been completed due to the lack of first-principles calculations of the surface stress tensor for a multi-component system with complex adsorbing species. One can, however, argue qualitatively that the adatom binding energy will alter in a periodic manner following the distribution of the dislocation network and, therefore, the surface strain modulation. It should be noted, that depending upon the nature of the surface stress tensor, the adatom binding energy could either increase or decrease with the strain and thus any qualitative representations should be viewed with this limitation in mind. In response to such a spatially-varying surface strain (Fig. 4c) and adatom binding energy (Fig. 4b), the film surface can evolve into a spatially-patterned, ordered array of multi-terrace islands (Fig. 4d) wherein 3D “wedding cake” structures are found to form in regions of the least compressive strain which gives rises to the highest binding energy (preference for strong adatom binding).

To confirm the presence of misfit dislocations in the heterostructure, transmission electron microscopy studies were completed on a heterostructure with a 55 nm thick PbZr_0.2_Ti_0.8_O_3_ film. Cross-sectional, dark field imaging taken under the **g** = (200) two beam condition along the [012] zone axis reveals the presence of edge-type misfit dislocations at the PbZr_0.2_Ti_0.8_O_3_/SrRuO_3_ interface (Fig. 4e). A magnified region of this image reveals contrast characteristic of the distortion of the lattice around the dislocation cores (Fig. 4f); in other words, we can directly image the strain fields produced by the dislocations. It can be seen that such a distortion extends close to the film surface and manifests as periodic strain oscillations as shown previously in the model above. By measuring several regions of the cross-section sample, the average strain modulation was found to be periodic with a period of ~200 nm corresponding to the surface ripple density of ~5 μm^−1^.

**Figure 5:**
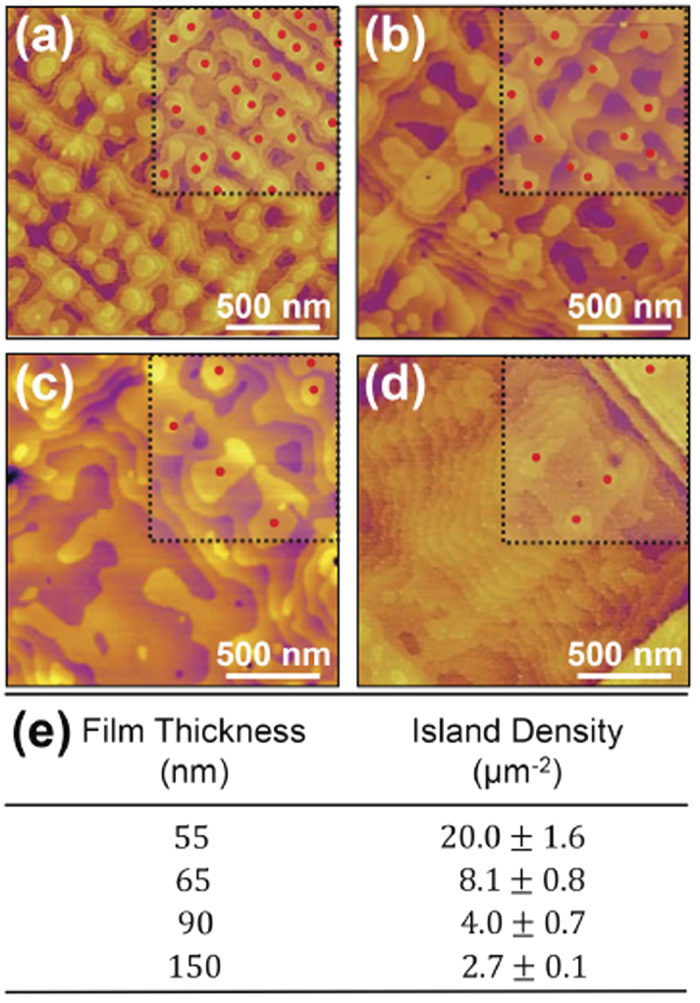
Atomic force microscopy images and illustration of island identification procedure for heterostructures with thicknesses of (**a**) 55 nm, (**b**) 65 nm, (**c**) 90 nm, and (**d**) 150 nm. (**e**) Measured island density as a function of increasing film thickness.

Having established a connection between the multi-terrace islands that form at the surface of the growing film and the misfit dislocations and strain modulations in the heterostructures, it is also important to understand how the growth process evolves as the strain is relaxed and the strain modulations subside (Regime II, Fig. 3). To do this, we focus on films in a thickness regime where dislocations have already formed and the multi-terrace islands are present to understand how further film growth drives a change in film morphology. In particular, we have extracted the top terrace island density from AFM studies of films varying from 55 nm to 150 nm in thickness to gain insight into the nature of island coarsening (Fig. 5a–d). It can be seen that the density of the top terrace islands decreases with increasing film thickness (Fig. 5e) indicating that as the strain is relaxed, the local variations in the binding energy are reduced, thus diminishing the preferential growth in specific strain-induced capture zones. As the binding energy profile is flattened across the surface, diffusion of mass from the islands to the valleys is enabled, thus resulting in coalescence of the islands. It should be noted, however, that despite a driving force for island-to-valley diffusion, the presence of the ES barrier restricts the extent to which surface diffusion can occur (as it restricts downward funneling) and thus prevents complete annihilation of island-like features and terraces on the surface. The net effect is, in turn, that the number of top-terrace islands increases (again, predominantly due to the ES barrier) while the islands themselves begin to coalesce.

## Discussion

**Figure 6:**
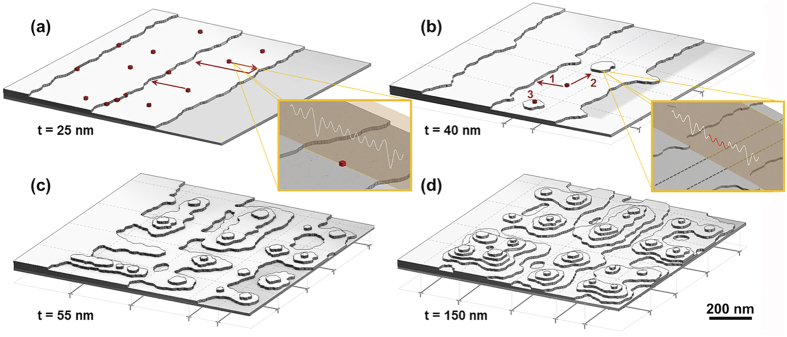
Schematic illustration of the thickness evolution of the surface structure. (**a**) In thin, unrelaxed films, step-flow growth persists; the arrows indicate the possibility of attachment of adatoms either directly to the step edges or after being reflected by the descending edge due to the ES barrier (inset shows the adatom potential energy landscape). (**b**) As the thickness increases, there is an onset of a kinetic instability due to dislocation formation and nucleation of islands in the capture zones (inset shows the adatom potential energy landscape is lowered, red color, due to the dislocation strain field). (**c**) With further increased film thickness, formation of an ordered array of dislocations induces ordered, multi-terrace island formation. (**d**) Continued film growth, leads to formation of multi-terrace islands and island coarsening.

The observed surface morphology evolution can be explained as follows (Fig. 6). In thin films (*i.e.*, 25 nm thick heterostructures) which have not relaxed, the surface in-plane strain resulting from the lattice misfit is spatially homogeneous enabling persistent step flow growth (Fig. 6a). As relaxation begins, the resulting strain modulations pattern the surface with an inhomogeneous adatom binding energy profile. As a result, there are local regions wherein there is stronger binding of the adatoms to the surface which, in turn, reduces the local desorption rate, gives rise to preferential growth in these specific capture zones, and disrupts the coherence of the step-flow growth (Fig. 6b). This is consistent with kinetic Monte Carlo simulations^41^ which suggest that periodic strain fields can alter the binding energy of the adatoms and experimental work on the growth of self-assembled Ge quantum dots which demonstrated that the quantum dots preferably grow first over the intersection of two perpendicular buried dislocations, second at a single dislocation line, and finally away from dislocations^42^. With subsequent deposition (*i.e.*, 40 nm thick heterostructures), the film can potentially grow in three different ways with adatoms 1) attaching to the existing vicinal step edges, 2) attaching at the edges around the freshly nucleated 2D islands, or 3) nucleating on top of the 2D island and growing as stable “wedding-cake” structures. AFM (Fig. 4d) confirms that the second and the third modes are preferred as a regular pattern of almost circular, multi-terrace islands grow in 2D periodic arrays. Once the surface is patterned with inhomogeneous strain, the vicinal steps do not advance as parallel edges and can grow outwards as finger-like protrusions thereby marking the transition from stable to unstable step-flow growth and finally ordered multi-terrace island formation (Fig. 6c). The fact that the islands are multi-terraced additionally suggests the existence of an ES barrier that inhibits down-step diffusion; adatoms that land on top of these islands can only desorb or nucleate as stable clusters that grow as a new terrace. With further growth, strain modulations on the surface are dampened with increasing thickness (Fig. 4a). Hence, the difference in the binding energy between the compressive strain maxima and minima decreases. In turn, the adatoms are less strongly confined to the capture zones, reducing nucleation and accentuating the coalescence of the existing islands (Fig. 5).

## Conclusions

The results presented here demonstrate that by gaining control of the nature of epitaxial strain relaxation, we are provided a route to produce self-patterned film surfaces as templates for fabrication of novel self-assembled nanostructures. Secondly, the preference of certain systems to produce defects along specific crystallographic directions provides a second level of control to produce ordered and periodic nanoscale structures. In summary, we showed that the strain fields from buried misfit dislocation arrays can strongly affect the dynamics of nucleation and growth in epitaxial thin films. A transition from 2D to 3D-island growth in PbZr_0.2_Ti_0.8_O_3_/SrRuO_3_/SrTiO_3_ (001) is observed with increasing film thickness. This transition is driven by the evolution of a periodic misfit-dislocation-induced strain field, in two orthogonal directions, that creates local binding energy variations which, in turn, give rise to local capture zones for preferential adatom nucleation. As the film thickness increases, a reduction in the surface strain modulation gives rise to a more homogeneous binding energy profile which, in turn, diminishes the influence of the local capture zones and results in a coalescence of the island features.

## Methods

To probe the evolution of surface morphology, PbZr_0.2_Ti_0.8_O_3_ (tetragonal, a = 3.95 Å and c = 4.13 Å)^43^ films of thickness 25–150 nm were grown on 25 nm SrRuO_3_/SrTiO_3_ (001) (cubic, a = 3.905 Å) substrates using pulsed-laser deposition. SrRuO_3_ was grown at 645 °C in an oxygen pressure of 100 mTorr with a laser fluence of 1.30 J/cm^2^ and frequency of 10 Hz. Subsequently, PbZr_0.2_Ti_0.8_O_3_ was grown at 635 °C in an oxygen pressure of 200 mTorr with a laser fluence of 1.35 J/cm^2^ and frequency of 3 Hz. SrRuO_3_ grows coherently strained to the substrate and the difference in lattice parameters of PbZr_0.2_Ti_0.8_O_3_ and SrTiO_3_ corresponds to a lattice mismatch of −1.24%. Detailed structural information for the various heterostructures was obtained using high-resolution X-ray diffraction (XPert MRD Pro equipped with a PIXcel detector, Panalytical) including θ–2θ scans and reciprocal space maps (RSMs). Topographic study of the films was carried out using atomic force microscope (AFM) (MFP-3D, Asylum Research). The cross-section, dark-field transmission electron microscopy (TEM) images of the heterostructures were obtained using JEOL 3010 microscope at the National Center for Electron Microscopy at Lawrence Berkeley National Laboratory.

## Additional Information

**How to cite this article**: Pandya, S. *et al.* Strain-induced growth instability and nanoscale surface patterning in perovskite thin films. *Sci. Rep.* **6**, 26075; doi: 10.1038/srep26075 (2016).
